# Genetic evidence that higher central adiposity causes gastro-oesophageal reflux disease: a Mendelian randomization study

**DOI:** 10.1093/ije/dyaa082

**Published:** 2020-06-26

**Authors:** Harry D Green, Robin N Beaumont, Andrew R Wood, Benjamin Hamilton, Samuel E Jones, James R Goodhand, Nicholas A Kennedy, Tariq Ahmad, Hanieh Yaghootkar, Michael N Weedon, Timothy M Frayling, Jessica Tyrrell

**Affiliations:** 1 Genetics of Complex Traits, University of Exeter Medical School, Exeter, UK; 2 IBD Pharmacogenetics, Royal Devon and Exeter NHS Foundation Trust, Exeter, UK

**Keywords:** GORD, UK Biobank, Mendelian randomization, adiposity, epidemiology, gastroenterology

## Abstract

**Background:**

Gastro-oesophageal reflux disease (GORD) is associated with multiple risk factors but determining causality is difficult. We used a genetic approach [Mendelian randomization (MR)] to identify potential causal modifiable risk factors for GORD.

**Methods:**

We used data from 451 097 European participants in the UK Biobank and defined GORD using hospital-defined ICD10 and OPCS4 codes and self-report data (*N* = 41 024 GORD cases). We tested observational and MR-based associations between GORD and four adiposity measures [body mass index (BMI), waist–hip ratio (WHR), a metabolically favourable higher body-fat percentage and waist circumference], smoking status, smoking frequency and caffeine consumption.

**Results:**

Observationally, all adiposity measures were associated with higher odds of GORD. Ever and current smoking were associated with higher odds of GORD. Coffee consumption was associated with lower odds of GORD but, among coffee drinkers, more caffeinated-coffee consumption was associated with higher odds of GORD. Using MR, we provide strong evidence that higher WHR and higher WHR adjusted for BMI lead to GORD. There was weak evidence that higher BMI, body-fat percentage, coffee drinking or smoking caused GORD, but only the observational effects for BMI and body-fat percentage could be excluded. This MR estimated effect for WHR equates to a 1.23-fold higher odds of GORD per 5-cm increase in waist circumference.

**Conclusions:**

These results provide strong evidence that a higher waist–hip ratio leads to GORD. Our study suggests that central fat distribution is crucial in causing GORD rather than overall weight.

**Key Messages**

Body mass index (BMI) is widely reported to associate with gastro-oesophageal reflux disease (GORD).
Previous studies on lifestyle factors such as alcohol and smoking have reported inconsistent findings.
Existing evidence for associations between obesity and lifestyle factors with GORD come from observational studies that are prone to confounding factors.
Our results show that the widely reported associations between GORD and BMI are confounded by measures of central adiposity.
We demonstrate that central adiposity is the more important causal risk factor for GORD.
These results suggest that clinicians should advise patients at risk of GORD to reduce their waist size.

## Introduction

Gastro-oesophageal reflux disease (GORD) has been defined as ‘symptoms or complications resulting from the reflux of gastric contents into the oesophagus or beyond, into the oral cavity (including larynx) or lung’.^1^ GORD is common, with typical symptoms of heartburn and acid regurgitation reported weekly by 13.3% of the general population.^2^

Numerous modifiable risk factors have been demonstrated to associate with GORD, but the majority of these have only been reported in observational studies, which are prone to confounding and reverse causality. For example, there is extensive observational evidence that adiposity [as measured by body mass index (BMI)] is associated with GORD. A large meta-analysis of 22 studies reports a 1.73-fold (1.46–2.06) increased risk of GORD in obese individuals (defined by BMI > 30 kg/m^2^)^2^ and a recent large genome-wide association study (GWAS) of GORD reports a genetic correlation between GORD and BMI.^3^ The literature also provides evidence that other measures of adiposity correlate with GORD phenotypes including waist circumference with reflux symptoms^4^ and waist–hip ratio (WHR) with both oesophageal inflammation (erosive reflux disease) and Barrett’s oesophagus.^5^ Several observational studies report an attenuation in the BMI association when BMI and WHR are included in multivariable models, suggesting that body-fat distribution may be an important factor in GORD.^6^

Patients commonly report that both alcohol and coffee consumption exacerbate GORD symptoms. However, a 2014 meta-analysis containing 15 case–control studies reported no significant association between GORD and coffee consumption,^7^ whereas the largest study included (*n* = 3153) reported a negative association.^8^ A recent (2019) meta-analysis of alcohol consumption and GORD reported conflicting evidence but suggested a positive association.^9^ Smoking is also frequently implicated in GORD, with studies demonstrating associations between GORD and (i) smoking duration and lifetime number of cigarettes smoked^8^ and (ii) both smoking history and current smoking status.^10^ Psychological factors such as stress^10^ and depression^11^ have both been reported to be associated with GORD, as well as miscellaneous factors such as wearing a belt too tightly,^12^^,^^13^ sugar intake,^14^ salt intake^8^ and heavy physical workload.^15^

**Figure 1:**
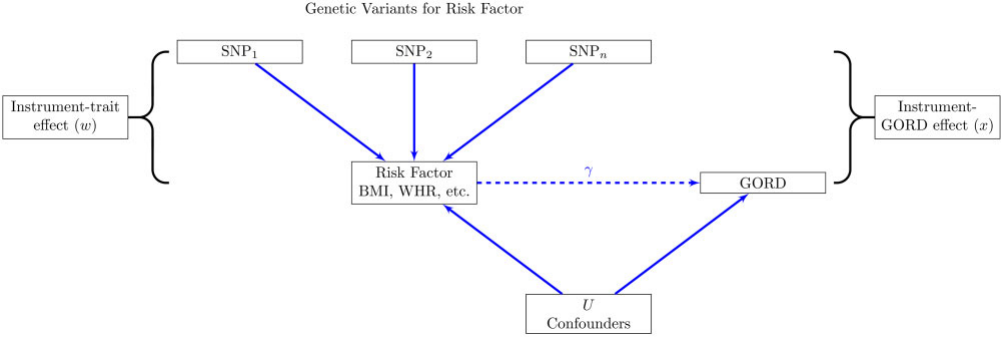
Principle of Mendelian randomization. If a specific risk factor [e.g. body mass index (BMI)] causes gastro-oesophageal reflux disease (GORD), genetic variants associated with that risk factor will also be associated with GORD. The genetic variants must not be associated with any other variables that could potentially confound the BMI–GORD association, such as lifestyle or environmental factors. Using the estimate of the genetic–BMI association ($\hat{w}$) and the apparent genetic–GORD association ($\hat{x}$), we can infer the causal effect of BMI on GORD ($\hat{y}=\hat{x}/\hat{w}$), which is expected to be free from confounding. If $\hat{y}$ is different to the observational associations, and assuming the core assumptions hold, this would suggest that the observational associations are confounded.

Mendelian randomization (MR; Figure 1) is a technique used to infer causal relationships between an exposure and an outcome by using genetic variants associated with the exposure. The variants associated with the exposure (e.g. BMI) can be used as an unconfounded proxy for the exposure, as their inheritance is random at conception. This method is now extensively used to infer causal pathways and has been used to study the impact of BMI on oesophageal adenocarcinoma and Barrett’s oesophagus.^16^ In this study, genetically predicted BMI was demonstrated to independently increase the risk of both oesophageal adenocarcinoma and Barrett’s oesophagus, but the power of the study was limited by the low numbers of cases. Furthermore, this study did not include other measures of adiposity such as WHR.

Here, we sought to assess the causal role of obesity measures [BMI, WHR, body-fat percentage (BFP) and a measure of higher but metabolically favourable adiposity] and lifestyle factors (smoking and caffeine) on the risk of GORD in up to 41 024 cases and 410 073 control individuals of European ancestry enrolled in the UK Biobank. First, we report observational associations to check for consistency with previous literature. Then, we used MR approaches to determine which of these associations have a likely causal role in GORD and which are potentially confounded at the observational level.

## Methods

### Participants

The UK Biobank is a large-scale study that aims to investigate the genetic and environmental basis of disease. Over 500 000 participants aged between 40 and 69 years were recruited between 2006 and 2010. Data collected include demographics, International Classification of Diseases 10th Revision (ICD10) hospital coding, medication records, anthropometric measures and a questionnaire containing lifestyle and mental-health factors. All participants have been genotyped, ∼450 000 using the Affymetrix Axiom UK Biobank array and ∼50 000 using the UK BiLEVE array. More detail on recruitment, demographics and data availability^17^ and on the collection and imputation of genomic data^18^ can be found elsewhere.

We defined 451 097 individuals of European descent using principal component analysis.^19^ We used well-imputed single nucleotide polymorphisms (SNPs) in the 1000 Genomes Cohort to project principal components from 1000 G and UK Biobank into the same space and clustered by the first four principal components to define Europeans. We also defined a subset of 379 713 unrelated individuals of European ancestry. Related individuals were defined using a KING Kinship^20^ to exclude those third-degree relatives or closer. An optimal list of unrelated individuals was generated by preferentially removing individuals with the maximum number of relatives to allow maximum numbers of individuals to be included; e.g. if A was related to B and C, but B and C were not, A was removed. For a simple pair, one individual was removed at random. Ancestral principal components were then generated within these identified individuals for use in subsequent analyses.

*Patient and public involvement*. This study uses data from the UK Biobank resource. Details of patient and public involvement in the UK Biobank are available online (https://www.ukbiobank.ac.uk/wp-content/uploads/2011/07/Summary-EGF-consultation.pdf? phpMyAdmin=trmKQlYdjjnQIgJ%2CfAzikMhEnx6). Patients were not specifically involved with setting the research questions and outcome measures or asked to contribute to the interpretation or publication of results. The results will not be directly disseminated to study participants, but the UK Biobank’s website contains summaries of key findings.

### GORD

We derived four GORD variables—one for use in primary analyses and three sensitivity analyses, each with increasing levels of certainty around the diagnosis of GORD. ICD10 and OPCS Classification of Interventions and Procedures version 4 (OPCS4) operation codes were obtained from the Hospital Episode Statistics (HES; https://digital.nhs.uk/data-and-information/data-tools-and-services/data-services/ hospital-episode-statistics). Self-reported conditions, risk factors and medication data were obtained from a verbal interviews at the UK Biobank Assessment Centre.

For the primary analysis, cases were defined as having any of the following:

a self-report code of 1138 (GORD/gastric reflux) in the non-cancer variable (n_20002_*);
an ICD10 code of K21.9 (GORD without esophagitis) or K21.0 (GORD with esophagitis) in HES;
an OPCS4 operation code of G24 (anti-reflux operations) or G25 (revision of anti-reflux operations) in the HES data.

All other individuals of European descent were included as controls for the primary analysis. This resulted in 33 969 cases vs 345 744 controls in unrelated individuals and 41 024 cases vs 410 073 controls in the related individuals. We also repeated this analysis using only the incident cases in the UK Biobank (10 664 cases vs 340 373 controls).

In Sensitivity Analysis 1, cases defined from only a self-reported definition of GORD were excluded. Controls taking medication common for reflux treatment (i.e. proton pump inhibitors and H2-receptor blockers) and controls who had had a diagnostic endoscopic examination of the upper gastrointestinal tract (OPCS4 code G45) were excluded due to the chance of these individuals having undiagnosed reflux. Sensitivity Analysis 1 contained 21 054 cases and 288 233 controls (25 372 cases and 340 996 in related individuals).

In Sensitivity Analysis 2, cases of GORD without esophagitis were excluded, leaving 10 135 cases and the same 288 233 controls.

**Figure 2:**
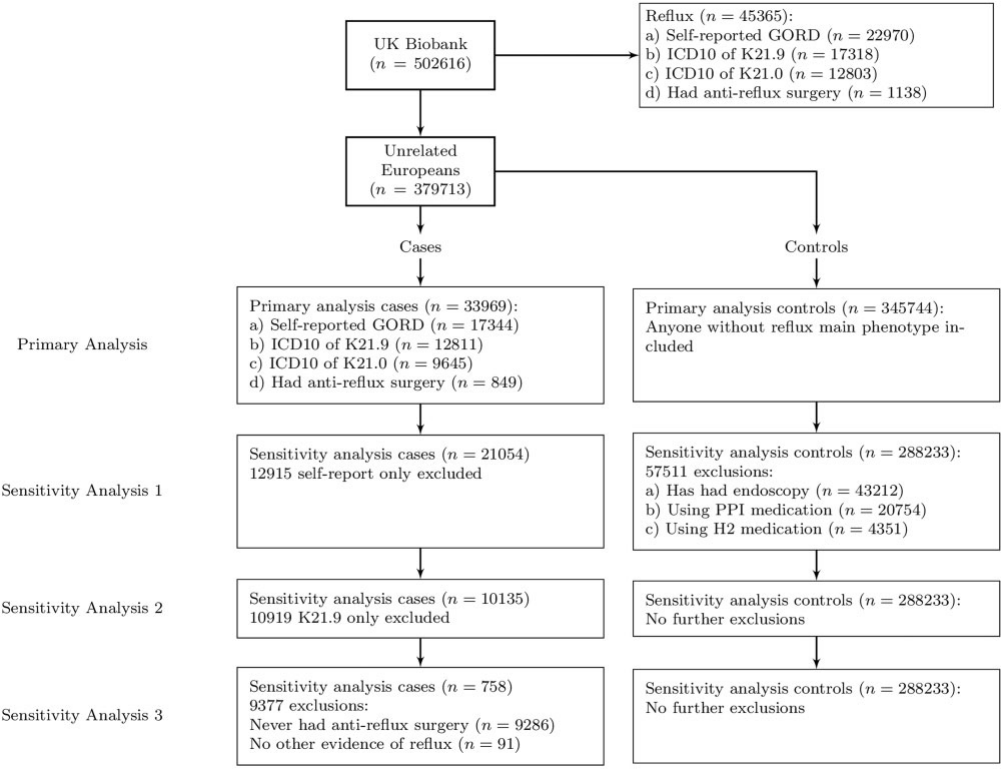
Definitions of gastro-oesophageal reflux disease (GORD). Flowchart showing definitions of phenotypes with numbers of people in each category for the 379 713 unrelated individuals in the UK Biobank, using ICD10 (International Classification of Diseases 10th Revision) codes, endoscopy status, proton pump inhibitor and H2-receptor blocker use to further refine the phenotype. The same flowchart for all 450 000 Europeans is given in Supplementary Figure 1, available as Supplementary data at *IJE* online. The farther down the sensitivity analysis, the greater the confidence we have that the cases are true GORD cases.

In Sensitivity Analysis 3, we kept only the cases that had undergone anti-reflux surgery, excluding those with no other confirmation of GORD, leaving only 758 cases and 288 233 controls. The exclusions for unrelated and related Europeans are summarized in Figure 2 and Supplementary Figure 1, available as Supplementary data at *IJE* online, respectively.

### Exposure variables

In this study, we investigated the role of seven exposure variables. The exposure variables are described briefly below:

*BMI—*BMI was calculated using the standard method of weight divided by height squared (kg/m^2^).
*WHR—*we used both a) WHR and b) WHR adjusted for BMI as measures of central adiposity. Both measures were derived as detailed by the GIANT consortium.^21^
*Coffee consumption—*coffee consumption was reported as number of caffeinated-coffee cups drunk per day, from a questionnaire. A coffee drinker in the observational analysis is defined as drinking >0 caffeinated-coffee cups per day.
*Cigarettes per day—*for former and current smokers, a pack-years variable was derived from the number of cigarettes smoked per day and the number of years an individual had smoked.
*BFP—*BFP was estimated by an impedance measurement by the UK Biobank.
*Favourable adiposity—*favourable adiposity was derived genetically and is detailed in the ‘Genetic variants’ section.

All continuous exposure variables were inverse-normalized prior to further analyses.

### Observational analysis

We utilized logistic-regression models to investigate the relationship between a range of demographic or predictor variables and GORD. We calculated the odds of GORD per unit change in the predictors and demographics. Age and sex were included as covariates in all models. These tests were repeated using Sensitivity Analyses 1 and 2 to check for directional consistency in a smaller but more well-defined cohort.

### Genetic variants

Imputation of genetic variants and associated quality control was performed centrally by the UK Biobank.^18^^,^^22^ For MR, genetic variants were selected from the UK Biobank imputation data set. Variants were excluded if imputation quality (INFO) was <0.9 or the minor allele frequency was <0.1%.

For each exposure trait (BMI, WHR, BFP, favourable adiposity, smoking and caffeine consumption), variants were selected based on reaching genome-wide significance (*P* < 5 × 10^−8^) from the largest available GWAS study of that trait. Where possible, exposure trait GWAS studies did not include the UK Biobank, as using variants discovered in the UK Biobank for our predictor traits would increase the possibility of statistical bias. The only exception was the genetic variants for favourable adiposity (variants that lead to increased weight but improved metabolic health) where effect sizes were unavailable for a non-UK Biobank analysis. Although we report waist circumference (WC) in the observational analyses, we do not report MR results for WC, because 21 of the 57 SNPs associated with WC adjusted for BMI were not independent of BMI.

*BMI.* The genetic variants extracted for BMI and our genetic risk score (GRS) for BMI utilized a 2015 GWAS of 339 224 individuals that reported 97 genome-wide significant loci.^23^ We excluded sex-specific variants and those with potential pleiotropy or secondary signals within a locus and utilized 72 variants.^24^

*WHR.* We used two different sets of variants and GRS for WHR, both derived from a meta-analysis of 694 649 individuals of European ancestry^25^:

382 SNPs associated with WHR;
463 SNPs associated with WHR adjusted for BMI.

*Favourable adiposity.* We used 14 variants for favourable adiposity, defined by SNPs that raise BFP but lower risk of metabolic disease. Further details on how these SNPs were identified is available elsewhere.^26^

*Caffeine coffee cups per day.* For caffeinated-coffee consumption, we used six SNPs from a 2015 genome-wide meta-analysis of habitual coffee consumption.^27^

*Cigarettes per day.* For smoking, we used four SNPs from a 2010 genome-wide meta-analysis of smoking behaviour.^28^

We did not include alcohol in the MR analysis due to the lack of availability of an appropriate genetic instrument.

The extracted genetic variants were used to create GRS for each potential GORD risk factor. Each variant in the GRS was weighted by its effect size $\left(\beta_{i}\right)$ on the risk factor, taken from the original GWAS to create a weighted score ${(W}_{s};$  Equation 1). $W_{s}$ was then rescaled by the number of variants (n) and the sum of $\beta_{i}$ values to calculate the weighted GRS ${(W}_{\mathrm{GRS}};$  Equation 2), which reflects the number of trait-raising alleles:

(1) $$W_{s}=\sum_{i=1}^{n}\beta_{i}\times SNP_{i}\;$$

 

(2) $$W_{\mathrm{GRS}}=\frac{W_{S}\times n}{\sum_{i=1}^{n}\beta_{i}}\;$$

All genetic instruments were associated with the relevant predictor, with high levels of statistical confidence and large F-statistics suggesting no weak instrument bias (in Sensitivity Analysis 3, we kept only those cases that had undergone anti-reflux surgery, excluding those with no other confirmation of GORD, leaving only 758 cases and 288 233 controls. The exclusions for unrelated and related Europeans are summarized in Figure 2 and Supplementary Table 2, available as Supplementary data at *IJE* online). Statistics showing potential pleiotropy between exposure traits are shown in Supplementary Table 3, available as Supplementary data at *IJE* online.

### MR

We used different MR methods to test the causal role of seven exposures on GORD. First, we performed standard one-sample instrumental variable analyses using the GRS in the unrelated data set of 379 713 individuals. Second, we investigated the causal relationship using a two-sample approach in the 451 097 related individuals. In this step, we explored whether our findings were robust to any potential influence of population stratification by using linear mixed models as implemented in the BOLT-LMM software.^29^ For both types of MR, the following assumptions must be met^30^:

- The genetic instruments used associate with the exposure trait with a high level of confidence. Here, we tested this assumption, using linear regression to explore the association of the GRS with the relevant predictors in the UK Biobank (Supplementary Table 2, available as Supplementary data at *IJE* online).
- The genetic instruments are independent of other factors that affect the outcome. This assumption is violated if (a) subgroups of the population have both different genotype frequencies and different distributions of the outcome or (b) the genetic variants used as instruments associate with confounders.
- The genetic instruments are linked with the outcome trait only through the respective exposure trait. This assumption is violated if the genetic instrument or another variant in high linkage disequilibrium has multiple (pleiotropic) effects.

#### One-sample MR: instrumental variable analysis

This analysis was performed in two stages. First, the association between the exposure and its GRS was assessed and the predicted values from this regression were saved. Second, the predicted values were used as the independent variable (reflecting an unconfounded estimate of variation in the exposure) and GORD was the dependent variable in a logistic-regression model. In these models, age, sex, ancestral principal components, assessment centre and genotyping platform were included as covariates. For WHR, we further estimated the causal influence of WC by using the correlation between the WHR adjusted for BMI GRS.

We performed a power calculation for our one-sample MR analysis using an online power calculator.^31^ Power to detect an association at alpha < 0.05 with an odds ratio greater than or equal to the observational result (Table 1) was 100% for BMI, WHR adjusted for BMI and WHR; moderate for BFP and favourable adiposity (81% and 63%); and limited for caffeine and smoking (5% and 13%) (Supplementary Table 2, available as Supplementary data at *IJE* online).

**Table 1:** Observational associations with gastro-oesophageal reflux disease (GORD)

Trait	Controls	GORD cases	*P*-value	Odds ratio (95% confidence interval)
*N*	345 744	33 969		
Male [*N* (%)]	159 150 (46.03%)	15 825 (46.59%)	0.67	1.00 (0.98–1.03)
Age (years) ± SD	57.04 ± 8.04	59.25 ± 7.4	<1 × 10^−15^	1.04 (1.04–1.04)
Townsend Deprivation Index ± SD	−1.5 ± 2.98	−1.22 ± 3.12	<1 × 10^−15^	1.11 (1.10–1.13)
Ever smoked [*N* (%)]	Ever: 153 287 (44.35%) Never: 187 836 (54.33%) Missing: 4621 (1.34%)	Ever: 17 076 (50.27%) Never: 16 383 (48.23%) Missing: 511 (1.5%)	<1 × 10^−15^	1.22 (1.19–1.25)
Current smokers [*N* (%)]	Smoker: 32 664 (9.45%) Non-smoker: 308 459 (89.22%) Missing: 4621 (1.34%)	Smoker: 3276 (9.64%) Non-smoker: 30 182 (88.85%) Missing: 511 (1.5%)	3 × 10^−7^	1.10 (1.06–1.15)
Cigarettes per day ± SD	18.4 ± 10.09	19.8 ± 10.91	<1 × 10^−15^	1.12 (1.10–1.14)
Caffeinated-coffee drinkers [*N* (%)]	Drinker: 221 046 (63.93%) Non-drinker: 71 128 (20.57%) Missing: 53 570 (15.49%)	Drinker: 19 971 (58.79%) Non-drinker: 8093 (23.82%) Missing: 5905 (17.38%)	<1 × 10^−15^	0.79 (0.77–0.81)
Caffeinated-coffee cups per day ± SD	2.63 ± 2.05	2.64 ± 2.12	0.003	1.02 (1.01–1.04)
Body mass index (kg/m^2^) ± SD	27.26 ± 4.74	28.59 ± 4.93	<1 × 10^−15^	1.28 (1.27–1.29)
Waist–hip ratio ± SD	0.87 ± 0.09	0.89 ± 0.09	<1 × 10^−15^	1.43* (1.41–1.46)
Waist–hip ratio (males) ± SD	0.87 ± 0.09	0.95 ± 0.07	<1 × 10^−15^	1.36 (1.33–1.39)
Waist–hip ratio (females) ± SD	0.81 ± 0.07	0.84 ± 0.07	<1 × 10^−15^	1.49 (1.46–1.52)
Waist circumference ± SD	89.95 ± 13.51	93.68 ± 13.07	<1 × 10^−15^	1.33 (1.31–1.36)
Body-fat percentage ± SD	31.12 ± 8.48	33.26 ± 8.69	<1 × 10^−15^	1.53 (1.50–1.55)

Table showing statistical differences in demographics between our primary GORD definition and controls in the UK Biobank. All *p*-values and odds ratios were calculated adjusted for age and sex. Cigarettes per day and caffeinated-coffee units were only defined in smokers and coffee drinkers. *N* for continuous variables: Coffee—241 016; Smoking—122 363; Townsend Deprivation Index—379 245; body mass index—378 214; waist–hip ratio—378 974; waist–hip ratio (male)—174 633; waist–hip ratio (female)—204 341; waist circumference—379 049; body-fat percentage—372 848. *when additionally adjusted for body mass index, OR = 1.29 (1.27–1.31).

#### Two-sample MR

A two-sample MR approach was utilized in the larger related subset of individuals, corrected for relatedness using BOLT-LMM v2.3, which accounts for population structure as part of the model.^29^ We extracted the variants for our seven exposures from the BOLT-LMM GWAS of the four definitions of GORD. We then performed inverse variance weighted (IVW) instrumental variable analysis and two further methods that are more robust to potential violations of the standard MR assumptions (MR-Egger^32^ and weighted-median MR^33^). The two-sample approach regresses the effect sizes of variant–outcome associations against the effect sizes of the variant–risk factor associations. IVW assumes no horizontal pleiotropy (under a fixed-effects model) or, if implemented under a random-effects model after detecting heterogeneity among the causal estimates, that (i) the strength of the association of the genetic instruments with the risk factor is not correlated with the magnitude of the pleiotropic effects and (ii) the pleiotropic effects have an average value of zero.

In contrast, the MR-Egger uses a weighted regression with an unconstrained intercept, thus removing the assumption that all genetic variants are valid instruments. Hence, this method is less susceptible to potentially pleiotropic variants. Weighted-median MR is also more resistant to pleiotropy. This method provides a consistent estimate of the causal effect if ≥50% of the information comes from valid instrumental variables. Given these different assumptions, if all methods are broadly consistent, it strengthens our causal inference. The R code for the various two-sample methods is available in Bowden *et al.* 2015^32^ and 2016.^33^

## Results

### Observational associations

The demographics of GORD cases and controls are summarized in Table 1. Briefly, participants with GORD were older, more deprived, more likely to be current or former smokers and smoked more cigarettes per day than controls. Individuals with GORD were less likely to drink caffeinated coffee, although, among coffee drinkers, we see a positive association with caffeinated-coffee cups per day.

We observed strong observational associations with all adiposity-related variables: BMI, WHR, WC and BFP. Higher odds of GORD were noted for a 1-standard-deviation (SD) higher BMI [odds ratio (OR): 1.28, 95% confidence interval (CI): 1.27–1.29], WHR (OR: 1.43, 95% CI: 1.41–1.46), WC (OR: 1.33, 95% CI: 1.31–1.36) and BFP (OR: 1.53, 95% CI: 1.50–1.55). These observational results were consistent when more stringent definitions of GORD were utilized (Supplementary Table 1, available as Supplementary data at *IJE* online).

### MR

#### MR of adiposity-related traits with GORD

**Figure 3:**
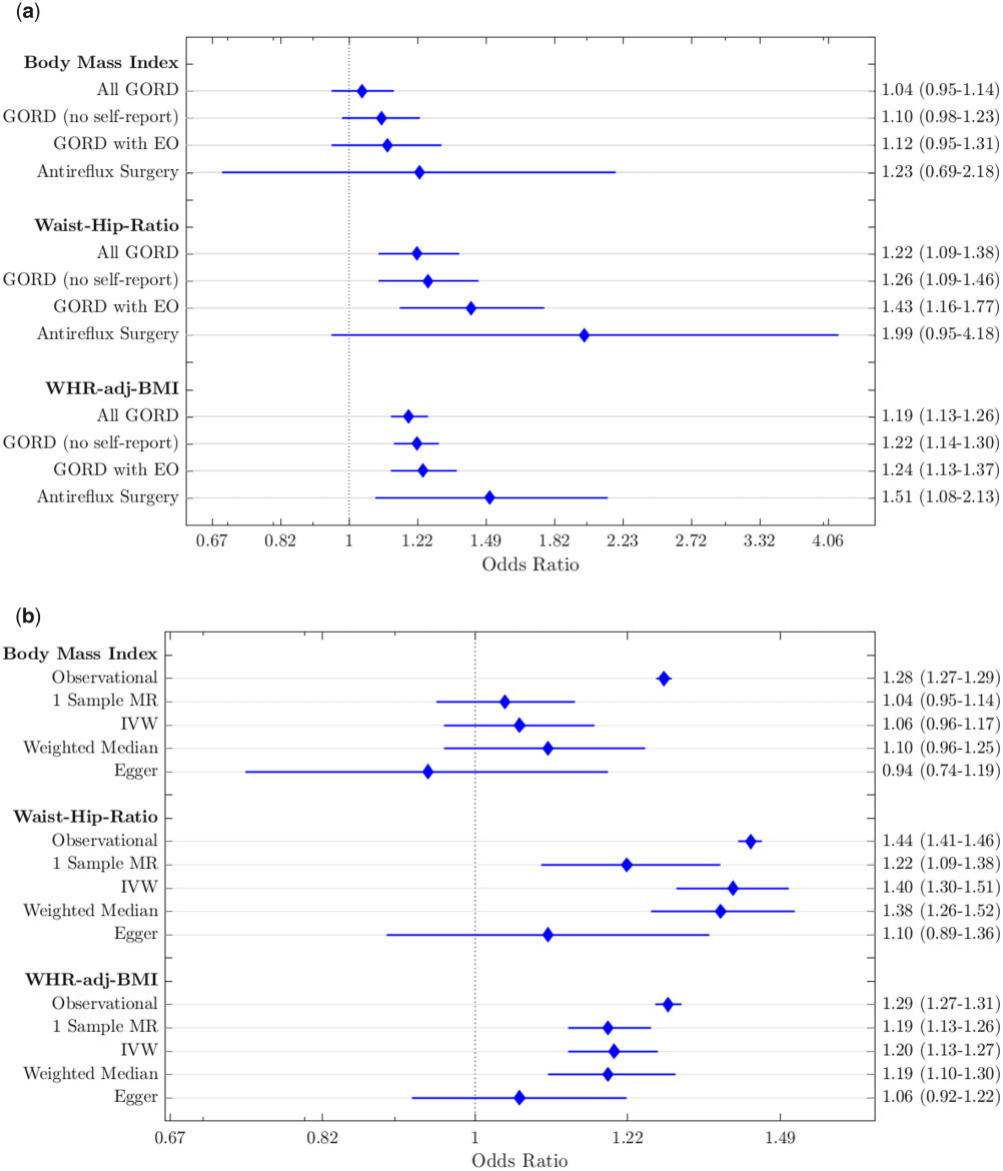
Forest plots of adiposity variables. (A) Comparison of analysis methods: showing the observational associations with gastro-oesophageal reflux disease (GORD), one-sample Mendelian randomization (MR) and two-sample MR associations (instrumental variable, weighted-median and Egger methods) for body mass index, waist–hip ratio and waist–hip ratio adjusted for body mass index in our primary analysis (all GORD). (B) Sensitivity analyses for the one-sample MR results showing similar results across all four analyses. On the right-hand axes are odds-ratio point estimates and 95% confidence intervals. All GORD, GORD (no self-report), GORD with erosive oesophagitis (EO) and Antireflux Surgery refer to primary Sensitivity Analyses 1, 2 and 3, respectively, described in Figure 2.

Genetic data provided evidence that a higher waist–hip ratio causes GORD with strong statistical confidence; a 1-SD higher WHR is associated with 1.22 higher odds of GORD (95% CI: 1.09–1.38; Table 2). Similar associations were noted when we used WHR adjusted for BMI, with a 1-SD higher WHR adjusted for BMI causing higher odds of GORD (OR: 1.19, 95% CI: 1.13–1.26; Table 2). These results were consistent when using the more stringent definitions of GORD (Figure 3A; Supplementary Table 4, available as Supplementary data at *IJE* online) and when using only the incident GORD cases (Supplementary Table 5, available as Supplementary data at *IJE* online). The two-sample MR approaches were directionally consistent (Figure 3B and Supplementary Table 6, available as Supplementary data at *IJE* online) with limited evidence of pleiotropy from the MR-Egger method (pintercept > 0.05, Supplementary Table 5, available as Supplementary data at *IJE* online). The WHR adjusted for BMI association equates to ∼1.19-fold higher risk of GORD per 4.2-cm larger WC (1.23-fold per 5 cm) using correlations between the WHR adjusted for BMI GRS and WC (Supplementary Table 7, available as Supplementary data at *IJE* online).

**Table 2:** Estimates of associations between risk factors and Gastro-oesophageal Reflux Disease (GORD) using Mendelian randomization

Exposure	*N*	Odds ratio	*p*-value
Caffeinated-coffee cups per day	241 016	1.18 (0.88–1.58)	0.28
Cigarettes per day	112 363	1.20 (0.84–1.72)	0.32
BMI	378 214	1.04 (0.95–1.14)	0.36
WHR adjusted for BMI	378 974	1.19 (1.13–1.26)	4.5 × 10^−11^
WHR	378 090	1.22 (1.09–1.38)	7.2 × 10^−4^
BFP	372 848	1.14 (0.94–1.38)	0.17
Favourable adiposity	372 848	1.10 (0.87–1.39)	0.43

Results of Mendelian randomization within the UK Biobank to show the causal effect of coffee, smoking, body mass index (BMI), waist–hip ratio (WHR), body-fat percentage (BFP) and favourable adiposity on GORD. Odds ratios are reported per standard deviation increase in the exposure trait. Coffee and cigarette analyses were performed only for drinkers/smokers. Sensitivity analyses are given in Supplementary Table 4, available as Supplementary data at *IJE* online.

Genetic data provided limited evidence that higher BMI (OR: 1.04, 95% CI: 0.95–1.14), BFP (OR: 1.14, 95% CI: 0.94–1.38) or favourable adiposity (OR: 1.10, 95% CI: 0.87–1.39) causes GORD (Table 2). The 95% CIs for BMI do not cross the observational result, suggesting the observational result is confounded. These results were consistent when the more refined phenotypes were used (Supplementary Table 4, available as Supplementary data at *IJE* online, Figure 3 and Supplementary Figure 2, available as Supplementary data at *IJE* online) and when only the incident cases were included (Supplementary Table 5, available as Supplementary data at *IJE* online). The results from the more pleiotropy-resistant methods were directionally consistent for BFP, but not for BMI (Supplementary Table 5, available as Supplementary data at *IJE* online), where the point estimate for Egger was in the opposite direction.

#### MR for the role of smoking and coffee drinking in GORD

We also found limited evidence that smoking (OR: 1.20, 95% CI: 0.84–1.72; Table 2) or caffeine consumption (OR: 1.18, 95% CI: 0.88–1.58; Table 2) causes GORD. These results were consistent with the more refined definitions of GORD (Supplementary Table 4, available as Supplementary data at *IJE* online). The point estimates from the MR analyses for the odds of GORD for both smoking and caffeine consumption were stronger than the respective observational results and directionally consistent.

## Discussion

### Principal findings

This study tested the causal role of adiposity and lifestyle-related exposures on GORD. First, we confirmed observational links between various adiposity measures (BMI, WC, WHR and BFP) and lifestyle factors (smoking and caffeine) with GORD in a large European population. Second, we used MR to provide evidence that higher WHR causes GORD, whilst there was little evidence for higher BMI causing GORD. We demonstrated that a 5-cm larger WC causes 1.23 higher odds of GORD. In contrast, for BMI, we could exclude an OR effect of >1.15 per 5 kg/m^2^ higher BMI. This contrasts with observational studies in which BMI is strongly associated with GORD.^2^^,^^34–36^ This study suggests that the observed relationship between higher BMI and GORD is principally driven by fat distribution, and central adiposity is a crucial factor.

Our MR results for BMI challenge the commonly held belief, inferred from observational studies and meta-analyses, that higher BMI causes GORD.^2^^,^^34–36^ The MR results were consistent with previous multivariate analyses that highlighted the importance of WHR in both erosive esophagitis and Barret’s Oesophagus. For example, in a small Korean population (500 cases), BMI was shown to be not associated with erosive esophagitis in multivariable models including WHR^5^ and, in a study of Barrett’s oesophagus (*N* = 193), the BMI association was greatly attenuated, whereas WHR remained strong.^6^ Our findings in a large European population provide strong evidence for the role of WHR in GORD. ORs were similar (within the 95% CI) when using only the incident cases and we still observe a Bonferonni significant *p*-value for WHR adjusted for BMI, but not for BMI (Supplementary Table 6, available as Supplementary data at *IJE* online).

The pathophysiology of GORD is complex and a number of factors have been implicated, including diminished gastric volume, increased intragastric pressure and decreased lower oesophageal sphincter pressure (secondary to chronic antral compression/displacement).^37^ Arguably, all of the factors are more feasibly driven by central adiposity rather than overall body weight. Our results are consistent with the proposed mechanisms behind waist-belt compression causing GORD by impairing oesophageal clearance,^12^ the increased incidence of reflux during later trimesters of pregnancy and variable GORD-symptom improvement following surgical weight-loss procedures despite BMI reduction.^38^

Whereas BMI is a commonly used and simple-to-calculate clinical measure, its use is limited by an inability to differentiate between fat and muscle mass.^39^ Additionally, as BMI may change without an alteration in the abdominal-fat distribution, our findings strongly support WHR, or more simply monitoring WC, as a more clinically useful measure for stratifying obesity-related GORD over BMI.

This study provided little evidence for a causal role for either smoking or caffeine consumption in GORD. However, these results were limited by the availability of only a small number of variants strongly and specifically associated with smoking heaviness and caffeine consumption. However, for both smoking (cigarettes per day) and caffeinated-coffee cups per day, the 95% CIs crossed both the respective observational point estimate and the null hypothesis. This does not provide robust evidence of either an association or confounding at the observational level or in previous research.^8^

### Strengths and weaknesses

One of the major strengths of this study was our large sample size, including ≤451 097 individuals, with 41 024 cases of GORD. This was similar in size to a recent meta-analysis exploring GORD risk factors, which included a pooled population of 460 984 and a prevalence of 13.3% (approximately 61 310 cases).^2^ The linked health data in the UK Biobank enabled us to perform sensitivity analyses with stricter case definitions than in the meta-analysis (GORD vs weekly reflux symptoms) and explore the associations with risk factors in much larger numbers (only 22/108 studies in the meta-analysis reported BMI, whilst 33/108 reported smoking status). This is the first study to perform MR to assess the causality of reported GORD risk factors and, with the sample size available, provided sufficient power to provide strong evidence for a likely causal role of higher WC rather than overall BMI on GORD.

We acknowledge some limitations with the study design. This study uses the UK Biobank data, which were collected from individuals aged between 37 and 73 years living in the UK with a bias towards healthy individuals. As such, the results of this study may not be generalizable to other age ranges. Further, we stratified the data to only include people of European ancestry, so results may not be generalizable to other ethnic groups. Supplementary Table 3, available as Supplementary data at *IJE* online, demonstrates that pleiotropy may be a confounding factor for the one-sample MR analysis; e.g. the BMI GRS associates with smoking and WHR adjusted for BMI associates with BFP. However, our main results are consistent when using pleiotropy-resistant two-sample MR methods (MR-Egger and IVW) and we observe no significant *p*-values for the MR-Egger intercept for either BMI or WHR adjusted for BMIs. Our primary analysis used self-reported GORD status and so is potentially unreliable, although our results were consistent across all sensitivity analyses, including those using disease-coding status from hospital records (ICD10). Finally, as our MR analysis relies on robust genetic associations with the risk factor, we were limited in what we could study. Whilst the WHR and BMI genetic variants were strong instruments for MR, achieving 100% power to detect an association with the observational odds ratio at *p* < 0.05, the genetic variants for smoking and caffeine were weaker and therefore these analyses were underpowered.

### Implications

Given the increasing prevalence of obesity in younger people, understanding the role of lifelong exposure to higher adiposity in GORD is crucial. Here, we have demonstrated that the most important adiposity-related risk factor for GORD is not body weight, but fat distribution.

Our results highlight the importance of a healthy body shape to reduce the risk of GORD and the importance of recording WHR when studying GORD. We provide evidence that, for individuals with GORD attempting to alleviate symptoms by losing weight, a reduction in WC is a better measure of progress than loss of overall body weight. In light of these results, more research is needed on the causal role of WHR in other related conditions.

## Supplementary data

Supplementary data are available at *IJE* online.

## Author contributions

H.D.G., T.A., M.N.W., T.M.F. and J.T. designed the study and wrote the manuscript. J.R.G. and N.A.K. edited the manuscript and helped to interpret the data. H.D.G., R.N.B., A.R.W., B.H., S.E.J., H.Y., M.N.W., T.M.F. and J.T. were involved in data processing, statistical analyses and interpretation. J.T. is the guarantor. All authors assisted in the writing, reviewing and approval of the manuscript.

## Funding

S.E.J. is funded by the Medical Research Council (grant: MR/M005070/1). A.R.W., T.M.F and H.Y. are supported by the European Research Council grants: SZ-245 50371-GLUCOSEGENES-FP7-IDEAS-ERC and 323195. H.Y. is also funded by the Diabetes UK RD Lawrence fellowship (grant: 17/0005594). R.N.B. is funded by the Wellcome Trust and Royal Society, grant 104150/Z/14/Z. J.T. is supported by an Academy of Medical Sciences (AMS) Springboard award, which is supported by the AMS, the Wellcome Trust, GCRF, the Government Department of Business, Energy and Industrial strategy, the British Heart Foundation and Diabetes UK (SBF004\1079). N.A.K. declares personal fees from Falk, Takeda and Pharmacosmos; other fees from Janssen; and non-financial support from Janssen, AbbVie and Celltrion outside the submitted work. J.R.G. received honoraria from Falk, AbbVie and Shield therapeutics, outside the submitted work for unrelated topics. T.A. reports grants from AbbVie, MSD, Napp Pharmaceuticals, Celltrion, Pfizer, Janssen and Celgene during this study; personal fees and non-financial support from Immunodiagnostik; personal fees and non-financial support from Napp Pharmaceuticals, AbbVie and MSD; personal fees from Celltrion and Pfizer; grants and personal fees from Takeda; and grants and non-financial support from Tillotts, outside the submitted work.

## Supplementary Material

dyaa082_Supplementary_Data
