# Supplementary material for: Genetic evidence that higher central adiposity causes gastro-oesophageal reflux disease: a Mendelian randomization study
Source: Int J Epidemiol. 2020 Jun 26;49(4):1270–81. doi: 10.1093/ije/dyaa082 (PMC7750946; doi:10.1093/ije/dyaa082)
Supplement: dyaa082_Supplementary_Data [file dyaa082_supplementary_data.pdf]

Supplementary Material

Figure S1

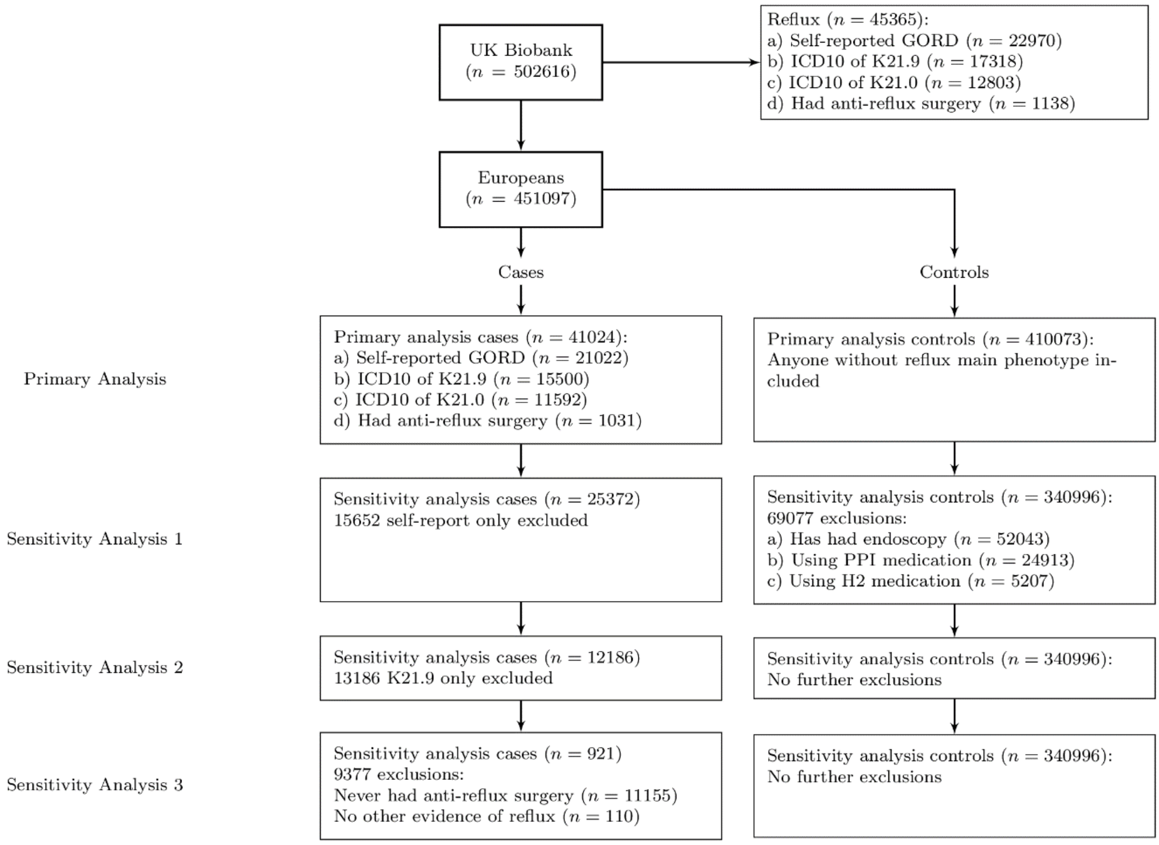

Supplementary Figure 1 - Flowchart showing definitions of phenotypes with numbers of people in each category of gastroesophageal reflux disease (GORD) for the 451,097 Europeans in the UK Biobank, using ICD10 (International Classification of Diseases 10th Revision) codes, endoscopy records from Hospital Episode Statistics, Proton Pump Inhibitor (PPI) and and H2 receptor blocker use to further refine the phenotype. The further down the Sensitivity analysis, the greater the confidence we have that the cases are true GORD cases.

Figure S2

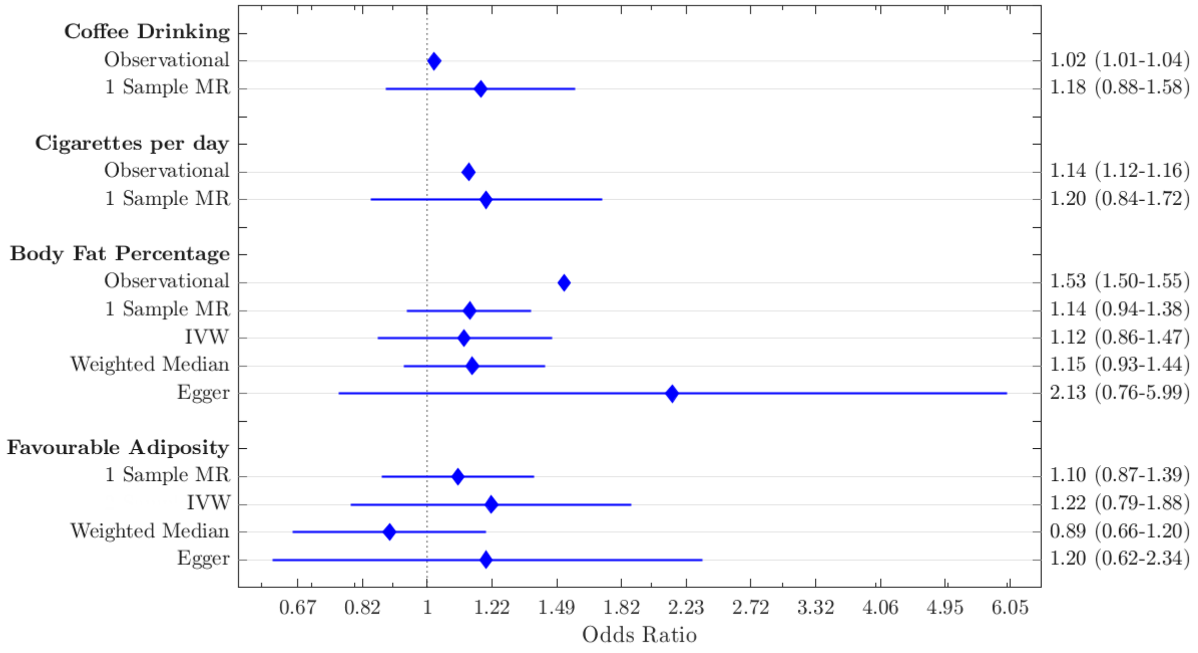

Supplementary Figure 2 - Comparison of analysis methods: showing the observational, 1 sample Mendelian randomisation (MR), and 2 sample Mendelian randomisation associations (instrumental variable analysis (IVW), weighted median and Egger) for coffee drinking, cigarettes smoked per day, body fat percentage and favourable adiposity in our primary analysis. There are no observational estimates for favourable adiposity, because this phenotype of higher adiposity and lower risk of disease can only be tested in this context with genetic variants.

Table S1

Trait	Controls	Cases	p-value	Odds Ratio
		Sensitivity Analysis 1		
**Male Gender**	46.6% [135,089/289,866]	45.58% [9,597/21,054]	0.004	0.96 (0.93-0.99)
**Age (years)**	56.59 +/- 8.07	59.44 +/- 7.33	0	1.05 (1.05-1.05)
**Townsend Deprivation Index**	-1.58 +/- 2.92	-1.13 +/- 3.15	$<1\times10^{-15}$	1.19 (1.17-1.21)
**Ever Smoked**	43.7% [125,041/286153]	51.91% [10,771/20,749]	$<1\times10^{-15}$	1.32 (1.28-1.36)
**Current Smoker**	14.12% [26,484/187,595]	17.72% [2,149/12,127]	$<1\times10^{-15}$	1.40 (1.34-1.47)
**Cigarettes Per Day**	18.09 +/- 9.81	19.96 +/- 11.22	$<1\times10^{-15}$	1.20 (1.19-1.22)
**Caffeinated Coffee Drinkers**	80.05% [232,029/289,866]	75.57% [15,910/21,054]	$<1\times10^{-15}$	0.73 (0.70-0.75)
**Caffeinated Coffee Per Day**	2.62 +/- 2.01	2.66 +/- 2.14	2.5×10^{-5}	1.04 (1.02 - 1.06)
**Body Mass Index (kg/m^2)**	27.11 +/- 4.64	28.6 +/- 4.99	$<1\times10^{-15}$	1.33 (1.31-1.35)
**Waist-Hip Ratio**	0.87 +/- 0.09	0.89 +/- 0.09	$<1\times10^{-15}$	1.52 (1.49-1.55)*
**Waist Circumference**	89.56 +/- 13.37	93.68 +/- 13.19	$<1\times10^{-15}$	1.41 (1.39-1.44)
**Body fat Percentage**	30.8 +/- 8.4	33.37 +/- 8.73	$<1\times10^{-15}$	1.60 (1.57-1.63)
		Sensitivity Analysis 2		
**Male Gender**	46.61% [136,059/291,905]	50.83% [5,152/10,135]	6.3e-17	1.18 (1.14-1.23)
**Age (years)**	56.61 +/- 8.07	59.45 +/- 7.31	2.7e-266	1.05 (1.05-1.05)
**Townsend Deprivation Index**	-1.58 +/- 2.92	-1.10 +/- 3.18	$<1\times10^{-15}$	1.20 (1.17-1.22)
**Ever Smoked**	43.75% [126,085/288,176]	52.24% [5,209/9,972]	$<1\times10^{-15}$	1.31 (1.25-1.36)
**Current Smoker**	14.15% [26,710/188,801]	18.43% [1,076/5,839]	$<1\times10^{-15}$	1.43 (1.33-1.53)
**Cigarettes Per Day**	18.10 +/- 9.82	20.39 +/- 11.99	$<1\times10^{-15}$	1.19 (1.17-1.21)
**Caffeinated Coffee Drinkers**	80.03% [233,609/291,905]	75.43% [7,645/10,135]	$<1\times10^{-15}$	0.71 (0.68-0.75)
**Caffienated Coffee Cups Per Day**	2.62 +/- 2.01	2.67 +/- 2.23	0.002	1.04 (1.01 - 1.07)
**Body Mass Index (kg/m^2)**	27.12 +/- 4.64	28.53 +/ 4.72	$<1\times10^{-15}$	1.30 (1.28-1.33)
**Waist-Hip Ratio**	0.87 +/- 0.09	0.90 +/- 0.08	$<1\times10^{-15}$	1.54 (1.50-1.58)*

**Waist Circumference**	89.59 +/- 13.38	94.21 +/- 12.54	$<1\times10^{-15}$	1.40 (1.37-1.43)
**Body fat Percentage**	30.81 +/- 8.4	32.71 +/- 8.75	$<1\times10^{-15}$	1.57 (1.53-1.62)

Observational associations for the first two sensitivity analyses. *when additionally adjusted for body mass index, odds ratio = 1.30 (1.27-1.34) in Sensitivity Analysis 1 and 1.40 (1.33-1.47) in Sensitivity Analysis 2.

Table S2

**GRS**	**F-stat**	**Power**	**R^2**
Caffeinated coffee per day	1066	0.05	0.0044
Cigarettes per day	503	0.13	0.0044
BMI	6267	1.00	0.0163
WHR adjusted for BMI	18066	1.00	0.0456
WHR	3473	1.00	0.0091
Body Fat Percentage	1310	0.81	0.0035
Favourable Adiposity	861	0.63	0.0023

Power calculations for genetic instruments, showing F statistic, power, and R^2. Odds ratios inputted were those of Table 1 – logistic regression adjusted for age and sex. Power is assuming a false positive rate of 0.05. R^2 values were calculated by regressing the genetic risk score (GRS) against its respective phenotype (body fat percentage for favourable adiposity). Acronyms: body mass index (BMI), waist hip ratio (WHR).

Table S3

**GRS**	**Coffee**	**Cigarettes**	**BMI**	**WHR_BMI**	**WHR**	**BFP**
Caffeinated coffee cups per day	β = 0.049 (0.046 – 0.052) $p<1\times10^{-15}$	β = 0.005 (0.002 – 0.008) p=0.003	β = 0.011 (0.008 – 0.014) $p=4.9\times10^{-12}$	β = 0.005 (0.001 – 0.008) p=0.005	β = 0.010 (0.007 – 0.013) $p=7.4\times10^{-10}$	β = 0.008 (0.005 – 0.012) $p=2.5\times10^{-7}$
Cigarettes per day	β = 0.004 (0.001 – 0.008) p=0.002	β = 0.037 (0.034 – 0.040) $p<1\times10^{-15}$	β = -0.001 (-0.005 – 0.002) p=0.38	β = 0.002 (-0.001 – 0.005) p=0.28	β = 0.000 (-0.003 – 0.003) p=0.86	β = 0.001 (-0.002 - 0.003) p=0.64
BMI	β = 0.020 (0.016 – 0.022) $p<1\times10^{-15}$	β = 0.009 (0.005 – 0.012) $p=2\times10^{-7}$	β = 0.128 (0.124 – 0.131) $p<1\times10^{-15}$	β = -0.007 (-0.011 – -0.004) $p=5.5\times10^{-6}$	β = 0.062 (0.059 – 0.065) $p<1\times10^{-15}$	β = 0.010 (0.097 – 0.103) $p<1\times10^{-15}$
WHR adjusted for BMI	β = -0.002 (-0.005 – 0.001) p=0.11	β = 0.006 (0.002 – 0.009) $p=7.4\times10^{-4}$	β = -0.034 (-0.037 – -0.031) $p<1\times10^{-15}$	β = 0.214 (0.211 – 0.217) $p<1\times10^{-15}$	β = 0.163 (0.160 – 0.166) $p<1\times10^{-15}$	β = -0.016 (-0.019 – -0.123) $p<1\times10^{-15}$
WHR	β = 0.000 (-0.003 – 0.003) p=0.89	β = 0.002 (-0.001 – 0.005) p=0.17	β = -0.023 (-0.026 – -0.020) $p<1\times10^{-15}$	β = 0.126 (0.123 – 0.128) $p<1\times10^{-15}$	β = 0.095 (0.092 – 0.098) $p<1\times10^{-15}$	β = -0.022 (-0.025 – -0.019) $p<1\times10^{-15}$
BFP	β = 0.011 (0.008 – 0.014) $p=1.2\times10^{-13}$	β = 0.003 (0.000 – 0.007) p=0.04	β = 0.066 (0.063 – 0.070) $p<1\times10^{-15}$	β = -0.005 (-0.008 – -0.001) 0.004	β = 0.031 (0.028 – 0.035) $p<1\times10^{-15}$	β = 0.060 (0.056 – 0.063) $p<1\times10^{-15}$
Favourable Adiposity	β = -0.005 (-0.005 – -0.002) p=0.002	β = 0.002 (-0.001 – 0.005) p=0.25	β = -0.018 (-0.021 – -0.014) $p<1\times10^{-15}$	β = 0.029 (0.026 – 0.033) $p<1\times10^{-15}$	β = 0.016 (0.013 – 0.019) $p<1\times10^{-15}$	β = -0.048 (-0.051 – -0.045) $p<1\times10^{-15}$

Association statistics (gradient, 95% CI, and p value) of a linear regression model of each genetic risk score (GRS) vs each exposure to check for potential pleiotropic effects. Acronyms: body mass index (BMI), waist hip ratio (WHR).

Table S4

Analysis	Exposure	Odds Ratio	p-value
**Primary Analysis**	Coffee Drinking	1.18 (0.88-1.58)	0.28
	Cigarettes per day	1.20 (0.84-1.72)	0.32
	BMI	1.04 (0.95-1.14)	0.36
	WHR adjusted for BMI	1.19 (1.13-1.26)	4.5e-11
	WHR	1.22 (1.09-1.38)	7.2e-4
	Body Fat Percentage	1.14 (0.94-1.38)	0.17
	Favourable Adiposity	1.10 (0.87-1.39)	0.43
**Sensitivity Analysis 1**	Coffee Drinking	1.13 (0.79-1.63)	0.50
	Cigarettes per day	1.13 (0.70-1.82)	0.62
	BMI	1.10 (0.98-1.23)	0.11
	WHR adjusted for BMI	1.22 (1.14-1.30)	1.1e-8
	WHR	1.26 (1.09-1.46)	2.3e-3
	Body Fat Percentage	1.13 (0.88-1.44)	0.34
	Favourable Adiposity	1.12 (0.83-1.51)	0.46
**Sensitivity Analysis 2**	Coffee Drinking	1.07 (0.65-1.77)	0.79
	Cigarettes per day	1.05 (0.53-2.08)	0.88
	BMI	1.12 (0.95-1.31)	0.16
	WHR adjusted for BMI	1.24 (1.13-1.37)	8.4e-6
	WHR	1.43 (1.16-1.77)	8.1e-4
	Body Fat Percentage	1.19 (0.84-1.68)	0.32
	Favourable Adiposity	1.24 (0.81-1.89)	0.33
**Sensitivity Analysis 3**	Coffee Drinking	5.38 (0.88-35.6)	0.08
	Cigarettes per day	1.97 (0.18-21.9)	0.58
	BMI	1.23 (0.69-2.18)	0.48
	WHR adjusted for BMI	1.51 (1.08-2.13)	0.02
	WHR	1.99 (0.95-4.18)	0.07
	Body Fat Percentage	2.71 (0.78-9.40)	0.17
	Favourable Adiposity	1.69 (0.36-7.95)	0.51

Sensitivity Analyses for 1 sample Mendelian Randomisation tests for the causal role of coffee, smoking, body mass index (BMI), waist hip raio (WHR), body fat percentage (BFP) and favourable adiposity on gastro-oesophageal reflux disease.

Table S5

Exposure	N	Odds Ratio	p-value
**BMI**	350,166	1.12 (0.96 to 1.30)	0.16
**WHR adjusted for BMI**	350,054	1.13 (1.03 to 1.24)	0.007
**WHR**	350,864	1.20 (0.98 to 1.46)	0.08

1 sample Mendelian Randomisation tests for the causal role of body mass index (BMI) and waist hip raio (WHR) on gastro-eosophageal reflux disease in only the incident cases in UK Biobank.

Table S6

Variable	Observational		Genetic: IVW		Genetic: Weighted Median		Genetic: Egger		
	Odds Ratio	p	Odds Ratio	p	Odds Ratio	p	Odds Ratio	p	p int
**BMI**									
**Primary**	1.28 (1.27 - 1.29)	$<1\times10^{-15}$	1.06 (0.96 - 1.17)	0.25	1.10 (0.96 - 1.25)	0.16	0.94 (0.74 - 1.19)	0.61	0.28
**Sensitivity 1**	1.33 (1.31 - 1.35)	$<1\times10^{-15}$	1.10 (0.98 - 1.23)	0.12	1.07 (0.93 - 1.24)	0.35	0.91 (0.68 - 1.20)	0.49	0.15
**Sensitivity 2**	1.30 (1.28 - 1.33)	$<1\times10^{-15}$	1.11 (0.96 - 1.28)	0.16	1.04 (0.85 - 1.28)	0.70	0.91 (0.64 - 1.29)	0.60	0.23
**Sensitivity 3**	1.27 (1.19 - 1.35)	2×10^{-13}	1.10 (0.71 - 1.70)	0.67	0.94 (0.47 - 1.87)	0.85	0.84 (0.29 - 2.42)	0.74	0.49
**WHR_BMI**									
**Primary**	1.29 (1.27-1.31)	$<1\times10^{-15}$	1.20 (1.13 - 1.27)	7.3×10^{-9}	1.19 (1.10 - 1.30)	4.1×10^{-5}	1.06 (0.92 - 1.22)	0.45	0.06
**Sensitivity 1**	1.35 (1.32-1.38)	$<1\times10^{-15}$	1.22 (1.13 - 1.31)	4.2×10^{-7}	1.23 (1.11 - 1.36)	5.3×10^{-5}	1.12 (0.94 - 1.34)	0.21	0.33
**Sensitivity 2**	1.41 (1.36-1.45)	$<1\times10^{-15}$	1.23 (1.11 - 1.37)	1.5×10^{-4}	1.23 (1.06 - 1.42)	0.006	1.27 (0.98 - 1.63)	0.07	0.81
**Sensitivity 3**	1.48 (1.33-1.64)	2×10^{-13}	1.37 (1.01 - 1.87)	0.05	0.94 (0.57 - 1.56)	0.81	1.14 (0.54 - 2.40)	0.74	0.59
**WHR**									
**Primary**	1.43 (1.41 - 1.46)	$<1\times10^{-15}$	1.40 (1.30 - 1.51)	$<1\times10^{-15}$	1.38 (1.26 - 1.52)	6×10^{-12}	1.10 (0.89 - 1.36)	0.36	0.02
**Sensitivity 1**	1.52 (1.49 - 1.55)	$<1\times10^{-15}$	1.49 (1.36 - 1.63)	$<1\times10^{-15}$	1.47 (1.31 - 1.64)	6×10^{-11}	1.04 (0.81 - 1.35)	0.74	0.004
**Sensitivity 2**	1.54 (1.50 - 1.58)	$<1\times10^{-15}$	1.51 (1.34 - 1.71)	2×10^{-10}	1.47 (1.25 - 1.72)	4.0×10^{-6}	1.38 (0.97 - 1.98)	0.08	0.60
**Sensitivity 3**	1.49 (1.36 - 1.62)	$<1\times10^{-15}$	2.06 (1.44 - 2.94)	8.7×10^{-5}	1.44 (0.81 - 2.56)	0.21	0.80 (0.29 - 2.22)	0.66	0.05
**BFP**									
**Primary**	1.53 (1.50 - 1.55)	$<1\times10^{-15}$	1.12 (0.86 - 1.47)	0.41	1.15 (0.93 - 1.44)	0.20	2.13 (0.76 - 5.99)	0.19	0.25
**Sensitivity 1**	1.60 (1.57 - 1.63)	$<1\times10^{-15}$	1.09 (0.83 - 1.44)	0.55	1.10 (0.85 - 1.42)	0.46	1.38 (0.42 - 4.51)	0.61	0.70
**Sensitivity 2**	1.57 (1.53 - 1.62)	$<1\times10^{-15}$	1.10 (0.79 - 1.54)	0.58	1.23 (0.87 - 1.73)	0.23	1.33 (0.32 - 5.52)	0.71	0.80
**Sensitivity 3**	1.66 (1.50 - 1.83)	$<1\times10^{-15}$	2.17 (0.57 - 8.20)	0.29	1.92 (0.53 - 6.99)	0.32	1.34 (0.00 - 406)	0.92	0.87
**Favourable Adiposity**									
**Primary**	NA	NA	1.06 (0.85 - 1.33)	0.60	0.89 (0.66 - 1.20)	0.45	1.20 (0.62 - 2.34)	0.59	0.70
**Sensitivity 1**	NA	NA	1.06 (0.82 - 1.38)	0.65	0.93 (0.65 - 1.34)	0.71	1.49 (0.70 - 3.15)	0.32	0.37
**Sensitivity 2**	NA	NA	1.12 (0.73 - 1.71)	0.62	1.15 (0.67 - 1.98)	0.60	1.69 (0.48 - 5.99)	0.43	0.51
**Sensitivity 3**	NA	NA	1.46 (0.41 - 5.27)	0.57	1.05 (0.18 - 6.04)	0.96	0.24 (0.01 - 9.81)	0.47	0.18

Sensitivity Analyses for 2 sample MR, showing the observational result, and three different methods for 2 sample MR: Inverse Variance Weighted (IVW), Weighted Median, and Egger. Exposures considered were body mass index (BMI), waist-hip ratio (WHR), body fat percentage (BFP) and favourable adiposity. Primary, and Sensitivity analyses are defined in Supplementary Table 1, and represent increasing certainty in cases. p values correspond to the odds ratio, p int is the p value for the intercept of the regression line in the Egger analysis.

Table S7

Phenotype	Beta (95% CI)	Standard Error	p-value
**Waist-Hip-Ratio**	0.001 (0.001 – 0.001)	1.4×10^{-5}	$<1\times10^{-15}$
**Waist Circumference**	-0.076 (-0.079 – -0.074)	0.0014	$<1\times10^{-15}$
**Hip Circumference**	0.043 (0.039 – 0.047)	0.0021	$<1\times10^{-15}$

Associations with the genetic risk score for waist-hip-ratio adjusted for body mass index with waist-hip-ratio, waist circumference and hup circumference
